# Risk factors for postoperative recurrence of ovarian endometriosis: long-term follow-up of 358 women

**DOI:** 10.1186/s13048-019-0552-y

**Published:** 2019-08-30

**Authors:** Xiao-Yan Li, Xiao-Pei Chao, Jin-Hua Leng, Wen Zhang, Jun-Ji Zhang, Yi Dai, Jing-Hua Shi, Shuang-Zheng Jia, Xiao-Xuan Xu, Si-Kai Chen, Yu-Shi Wu

**Affiliations:** Department of Obstetrics and Gynecology, Peking Union Medical College Hospital, Peking Union Medical College & Chinese Academy of Medical Science, Shuaifuyuan No. 1, Dongcheng District, Beijing, 100730 China

**Keywords:** Ovarian endometriomas, Recurrence, Risk factors

## Abstract

**Objective:**

To explore the risk factors for the recurrence of endometrioma and the risk factors for the recurrence of endometriosis-related pain after long-term follow-up.

**Methods:**

This study retrospectively analyzed 358 women with endometriomas who had a minimum of 5-years follow up after laparoscopic endometrioma excision, which was performed at Peking Union Medical College Hospital from January 2009 to April 2013. All women were divided into recurrence group and nonrecurrence group. Analysis was performed with regard to preoperative history, laboratory analysis, findings during surgery, and symptoms during follow-up, including improvement and recurrence.

**Results:**

The cumulative incidence rates of recurrence from 5 to 10 years after surgery were 15.4, 16.8, 19.3, 22.5, 22.5, and 22.5%, respectively. Significant differences were found between two groups in terms of age at surgery (RR: 0.764, 95% CI: 0.615–0.949, *p* = 0.015), duration of dysmenorrhea (RR: 1.120, 95% CI: 1.054–1.190, *p* < 0.001), presence of adenomyosis (RR: 1.629, 95% CI: 1.008–2.630, *p* = 0.046), CA125 level (RR: 1.856, 95% CI: 1.072–3.214, *p* = 0.021) and severity of dysmenorrhea. The severity of dysmenorrhea (RR: 1.711, 95% CI: 1.175–2.493, *p* = 0.005) and postoperative pregnancy (RR: 0.649, 95% CI: 0.460–0.914, *p* = 0.013) were significantly correlated with endometrioma recurrence in the multivariate analysis. No significant associations were found between the recurrence rate and gravida, parity, body mass index, infertility, leiomyoma presence, the size of ovarian endometrioma, the presence of deep infiltrating endometriosis, disease stage or postoperative medication.

**Conclusions:**

The severity of dysmenorrhea and postoperative pregnancy were independent risk factors for the recurrence of ovarian endometriomas after surgery during the long-time follow up.

## Introduction

Endometriosis is a chronic benign estrogen-dependent disease. It is observed primarily in patients of reproductive age, and its prevalence in this population is estimated to be 5–10%. Endometriosis is defined as the presence of active endometrial tissue outside the uterine cavity, usually on the peritoneum of the minor pelvis, ovaries and fallopian tubes and sometimes in extraperitoneal regions. Based on the locations of the lesions, the disease is classified as peritoneal, ovarian or deep infiltrating endometriosis [1, 2]. Endometriosis causes impaired quality of life (QoL) for women of reproductive age, and has malignant clinical manifestation despite being a benign disease. Ovarian endometriosis is the most common type, accounting 17% and 44% of all endometriosis. Laparoscopic conservative surgery has been considered the gold standard treatment for ovarian endometrioma [3, 4]. However, surgery may affect ovarian reserve function, and thus, surgery, especially repeat surgery, is not recommended for ovarian endometrioma recurrence [5–7]. The recurrence rate following surgical intervention remains high, even for those who receive postoperative medical therapy. Therefore, endometrioma recurrence is one of the most important unresolved problems in the management of endometriosis. The recurrence rate of ovarian endometrioma after conservative laparoscopic surgery has been reported to be 29–56% at 2 years and 43% at 5 years. When postoperative medical treatment was introduced, the endometrioma recurrence rate substantially declined to 3–11% at 2 years and 6% at 5 years [8, 9]. A pooled analysis of 23 studies estimated recurrence rates of 21.5% at 2 years and 40.0–50.0% at 5 years after primary surgery [10]. Unfortunately, few studies have analyzed the determinants of the long-term recurrence rate for endometrioma beyond 5 years after surgery. Therefore, the aim of this study was to explore the risk factors for endometrioma recurrence and the risk factors for the recurrence of endometriosis-related pain after long-term follow up.

## Materials and methods

### Ethical approval

This study was approved by the Ethics Committee of Peking Union Medical College Hospital. Written informed consent was obtained from all participants.

### Patient population

We collected 358 women with endometriomas who had a minimum of 5 years of postoperative follow-up after undergoing a laparoscopic cystectomy at Peking Union Medical College Hospital from January 2009 to April 2013.

The inclusion criteria were as follows: (1) the diagnosis was confirmed by pathologists; (2) ultrasonography was conducted to determine endometrioma recurrence at least 6 months after surgery; and (3) patients were observed without postoperative medications or were treated with postoperative gonadotropin-releasing hormone agonist (GnRHa) injections for 3–6 cycles, with or without a Mirena levonorgestrel-releasing intrauterine device (LNG-IUD). (4) The duration of follow-up was at least 5 years. The exclusion criteria were as follows: (1) age < 20 or > 45 years; (2) having underwent bilateral oophorectomy or hysterectomy; and (3) intra-operative conversion to laparotomy.

### Methods

Medical charts were reviewed to collect data on age at surgery; body mass index; presence of adenomyosis or leiomyoma; surgical history; symptoms of dysmenorrhea; parity; size of endometrioma; location of cysts; serum CA125 levels; operative time; intraoperative blood loss; American Society for Reproductive Medicine (ASRM) stage [11]; postoperative medications; postoperative pregnancy; and recurrence time. The size of the ovarian endometrioma was defined as the largest diameter of cysts. Obliteration of the pouch of Douglas was defined when there was any adhesion in the pouch.

All surgeries were performed by one expert laparoscopist. In all cases, the aim of the surgical procedures was to remove all visible implants of endometriosis, complete the opening of the pouch in the operation and perform lysis of adhesion. Endometriosis was staged according to the classification of the ASRM. The presence, localization, and extent of typical powder-burn and subtle lesions, adhesions, and deep infiltrating implants were recorded. Both ovaries were then completely mobilized. A sharp cortical incision was made into the cyst, and a cleavage plane was identified. A traumatic forceps and counter traction were used to strip the endometrioma from the ovarian parenchyma. Hemostasis was achieved by the selective application of bipolar coagulation. After the endometriomas were removed, all remaining visible endometriotic lesions were excised or fulgurated. Anatomical restoration was then achieved. Specimens underwent thorough histological analysis. When complications occurred, laparoscopy was converted into laparotomy. Laparotomic conversions were not included in the study.

In our center, all women are followed up according to an internal protocol. A standard gynecological examination and a transvaginal ultrasound are conducted before surgery; at 3, 6, and 12 months after surgery; and then yearly after surgery. Menstrual-reproductive factors and pain symptoms of pain are also evaluated. During the follow-up visits, patients were asked whether dysmenorrhea, pelvic pain or dyspareunia occurred during the follow-up period. The time of their first appearance and the intensity of the pain symptoms after laparoscopy were also documented. Pain was rated on the basis of a 10-cm visual analog scale (VAS), and the intensity was divided into none (0), mild (1–4), moderate (5–7), or severe (8–10). The presence of pain before surgery and pain recurrence/occurrence were defined as the recurrence of pain after a period of at least 3 months of relief after surgery. Threshold points defining different severities of pain were chosen based on a previous correlation analysis [12]. Endometrioma recurrence was determined using ultrasonography. Recurrent endometrioma was defined as the presence of a persistent ovarian cyst that had a thin wall (with a diameter of at least 2 cm), regular margins, a homogenous low echogenic fluid content with scattered internal echoes and did not resolve after several successive menstrual cycles. An improvement in pain was defined as a decrease in the VAS score for at least two points. Persistent pain was defined as no improvement or an increase in the VAS score of less than 2 points after surgery. Recurrence was identified when the same VAS score as that before surgery was noted at follow-up.

### Statistical analysis

Statistical analysis was performed using the Statistics Package for Social Sciences Version 22.0 (SPSS Inc., Chicago, IL, USA). Quantitative variables were compared using t-tests and ANOVA with Bonferroni’s correction for multiple testing. Fisher’s exact or chi-square tests were used to analyze qualitative variables. Potential risk factors (*p* < 0.2) were identified using univariate analysis and Cox’s multivariate proportional hazard analysis. The hazard ratio (HR) and 95% confidence interval (CI) were calculated as a measure of the risk of recurrence in each study. Significance was defined as *p* < 0.05.

## Results

For the total of 358 women, the mean age and BMI were 33.2 ± 5.4 years and 21.2 ± 2.6 kg/m^2^. The median gravida, parity, and duration of dysmenorrhea were 1(0–10), 0(0–2) and 24 months (0–360 months). The median CA125 level was 54 U/ml (8.4–754 U/ml). The mean operation time, bleeding volume and duration of follow-up were 66.4 ± 22.3 min, 5.4 ± 73.5 ml, and 84.2 ± 14.6 months. Before surgery, dysmenorrhea was absent in 23.7% (85/358) of cases, mild in 14% (50/358), moderate in 21.8% (78/358), and severe in 40.5% (145/358). The infertility rate before surgery was 18.4% (66/358). 17.9% (64/358) cases had concurrent leiomyoma, 53.1% (190/358) had concurrent deep infiltrating endometriosis, and 39.9% (143/358) had concurrent adenomyosis. Abdominal and vaginal ultrasound revealed the presence of bilateral ovarian endometriomas in 46.1% (165/358) of cases and unilateral ovarian endometriomas in 53.9% (193/358) of cases (31.0%, 111 left, 22.9%, 82 right).

At laparoscopy, ovarian endometrioma was confirmed with a mean size of 5.4 ± 2.1 cm on the left side and 5.3 ± 1.9 cm on the right. Leiomyomas were present in 18% (64/358) of cases, deep infiltrating endometriosis 53.1% (190/358), and adenomyosis 39.9% (143/358). One hundred two patients had partial and 162 patients had total cul-de-sac obliteration. According to the rASRM classification, 134 (37.4%) and 213 (59.5%) cases were stage III and stage IV. Postoperative therapy was chosen on the basis of individual characteristics and careful counseling with the patient: 12 (3.4%) women had no therapy; 12 (3.4%) oral contraceptives (drospirenone and ethinylestradiol tablets for at least 1 year); 14 (3.9%) Mirena for least 1 year, 242 (67.6%) received GnRHa for 3–6 months, and 78 (21.8%) had GnRHa followed by Mirena.

After more than 5-years follow-up (median 83, 60–120), we observed 9.4% (34/358) had pain recurrence, 6.4% (23/358) cyst recurrence and 3% (11/358) for both. The cumulative rate of endometrioma and/or endometriosis-related pain recurrence over 5 years was 19.0%. Sixty-eight women were in the recurrence group and 290 cases in the nonrecurrence group. Dysmenorrhea recurred in 34 women (68%), endometrioma recurred in 23 women (33.8%), and both recurred in 11 patients (16.2%). The clinical characteristics of the different groups of endometriomas are shown in Table 1.

**Table 1:** Baseline characteristics of the endometrioma and/or endometriosis-related pain recurrence and nonrecurrence groups

Characteristics	Recurrence group (*n* = 68)	Nonrecurrence group (*n* = 290)	χ^2^	*p* value
Age, y	31.8 ± 5.0	32.9 ± 5.2	1.457	0.146
Gravida	0 (0–4)	1 (0–10)	1.207	0.228
Parity	0 (0–1)	0 (0–2)	2.385	0.018
BMI, kg/m^2^	20.9 ± 2.5	21.2 ± 2.6	0.814	0.416
rAFS stage			3.093	0.377
Stage I	0 (0%)	1 (0.3%)		
Stage II	3 (4.4%)	7 (2.4%)		
Stage III	20 (29.4%)	113 (39.2%)		
Stage IV	45 (66.2%)	167 (58.0%)		
Largest-diameter endometrioma, cm				
Left	5.6 ± 1.9	5.4 ± 2.2	−0.707	0.481
Right	5.7 ± 2.0	5.2 ± 1.8	−1.631	0.104
CA125, U/ml	99.23 ± 89.72	100.37 ± 228.61	0.038	0.970
Dysmenorrhea, VAS	7 (0–10)	5 (0–10)	−3.018	0.003
Extent of dysmenorrhea			15.420	0.001
None	4 (5.9%)	81 (27.9%)		
Mild	10 (14.7%)	40 (13.8%)		
Moderate	20 (29.4%)	58 (20.0%)		
Severe	34 (50.0%)	111 (38.3%)		
Duration of dysmenorrhea, m	84 (0–360)	24 (0–348)	−3.514	0.001
Endometrioma side			3.082	0.214
Left	16 (23.5%)	95 (32.8%)		
Right	20 (29.4%)	62 (21.4%)		
Bilateral	32 (47.1%)	133 (45.9%)		
Obliteration of cul-de-sac			3.307	0.191
Absent	13 (19.1%)	81 (27.9%)		
Partial	18 (26.5%)	84 (29.0%)		
Complete	37 (54.4%)	125 (43.1%)		
Leiomyoma	18 (26.5%)	46 (16.0%)	5.057	0.044 (0.044)
Deep infiltrating endometriosis	41 (63%)	149 (51.4%)	1.758	0.224
Adenomyosis	34 (50.0%)	108 (37.4%)	4.059	0.044 (0.044)
Postoperative dysmenorrhea relief	55 (87.3%)	8 (90.0%)	1.770	0.413
Infertility			2.408	0.300
Primary	11 (16.2%)	32 (11.0%)		
Secondary	6 (8.8%)	17 (5.9%)		
Operation			0.705	0.590
Cystectomy	65 (95.6%)	21 (72%).		
Salpingo-oophorectomy	3 (4.4%)	269 (92.8%)		
Postoperative pregnancy	24 (35.3%)	113 (39.0%)	0.611	0.894
Postoperative medication			11.066	0.026
None	0 (0%)	12 (4.1%)		
OCP	1 (1.5%)	11 (3.8%)		
Mirena	1 (1.5%)	13 (4.5%)		
GnRHa with LNG-IUD	9 (13.2%)	69 (23.8%)		
GnRHa without LNG-IUD	57 (83.8%)	185 (63.8%)		
Duration of follow-up	83 (60–120)	85 (60–116)	−0.459	0.646

*Abbreviations*: *BMI* Body mass index, *CA-125* Cancer antigen 125, *cm* centimeter, *GnRHa* Gonadotropin-releasing hormone agonist, *LNG-IUD* Levonorgestrel-releasing intrauterine device, *m* month, *OCP* Oral contraceptive pills, *VAS* Visual analogue score

Univariate analysis and Cox’s multivariate proportional hazard analyses were performed. Differences were found between two groups in terms of age at surgery (RR: 0.764, 95% CI: 0.615–0.949, *p* = 0.015), duration of dysmenorrhea (RR: 1.120, 95% CI 1.054–1.190, *p* < 0.001), presence of adenomyosis (RR: 1.629, 95% CI: 1.008–2.630, *p* = 0.046), CA125 level (RR: 1.856, 95% CI: 1.072–3.214, *p* = 0.021) and extent of dysmenorrhea. The risk ratio of mild dysmenorrhea was 4.506 (95% CI: 1.413–14.368, *p* = 0.011), moderate was 2.451 (95% CI: 1.433–4.193, *p* = 0.001), and severe dysmenorrhea was 1.771 (95% CI: 1.254–2.502, *p* = 0.001).

No significant association was found between endometrioma and/or endometriosis-related pain recurrence rate and gravida, parity, BMI, infertility, leiomyoma presence, ovarian endometrioma size, deep infiltrating endometriosis presence, obliteration of the pouch of Douglas, disease stage and postoperative medication (Table 2). Only the extent of dysmenorrhea (RR: 1.711, 95% CI: 1.175–2.493, *p* = 0.005) and postoperative pregnancy (RR: 0.649, 95% CI: 0.460–0.914, *p* = 0.013) were significantly correlated with endometrioma and/or endometriosis-related pain recurrence in the multivariate analysis (Table 2).

**Table 2:** Univariate and multivariate analysis of risk factors in the endometrioma and/or endometriosis-related pain recurrence and nonrecurrence groups

Factor	Univariate analysis			Multivariate analysis		
	Relative risk	95%CI	*P* value	Relative risk	95%CI	*P* value
Age at surgery (per 5 years)	0.764	0.615–0.949	0.015	0.738	0.520–1.048	0.090
Gravida						
0	1.000					
1	0.673	0.370–1.224	0.194			
2 (and above)	0.757	0.557–1.029	0.075			
Parity						
0	1.000					
1	0.478	0.261–0.876	0.017	0.002	0.000–1.222	0.974
2	0.216	0.011–4.254	0.314			
BMI	0.820	0.478–1.407	0.470			
Infertility	1.527	0.882–2.644	0.131			
Extent of dysmenorrhea				1.711	1.175–2.493	0.005
None	1.000					
Mild	4.506	1.413–14.368	0.011			
Moderate	2.451	1.433–4.193	0.001			
Severe	1.771	1.254–2.502	0.001			
Duration of dysmenorrhea (per year)	1.120	1.054–1.190	< 0.001	1.026	0.944–1.114	0.548
Extent of dysmenorrhea	2.534	1.408–4.563	0.002			
VAS (0-4)						
VAS (5-10)						
Dyspareunia	1.734	1.049–2.866	0.032	1.081	0.511–2.285	0.839
Chronic pelvic pain	0.959	0.503–1.828	0.898			
Dyschezia	0.735	0.268–2.018	0.550			
Presence of leiomyoma	1.661	0.969–2.848	0.065	1.713	0.825–3.555	0.149
Presence of adenomyosis	1.629	1.008–2.630	0.046	1.446	0.719–2.908	0.301
Presence of deep infiltrating endometriosis	1.380	0.849–2.243	0.194	1.118	0.582–2.149	0.737
CA125 level (45 U/ml)	1.856	1.072–3.214	0.021	1.020	0.965–1.078	0.491
rARSM stage	1.204	0.774–1.873	0.409			
Bilateral involvement	1.046	0.650–1.684	0.853			
Large dimeter (per 10 mm)	1.016	0.944–1.093	0.674			
Postoperative medication						
None	1.000					
GnRHa	21.818	0.125–3810.382	0.242			
OCP	4.057	0.019–856.392	0.610			
Mirena	2.741	0.048–155.496	0.624			
GnRHa+Mirena	1.991	0.375–9.730	0.436			
Postoperative pregnancy	0.938	0.655–1.344	0.728	0.649	0.460–0.914	0.013

*Abbreviations*: *BMI* Body mass index, *CA-125* Cancer antigen 125, *GnRHa* Gonadotropin-releasing hormone agonist, *OCP* Oral contraceptive pills, *VAS* Visual analogue score

## Discussion

There are a variety of factors, both clinically and surgically, that might be related to the risk of endometrioma and/or endometriosis-related pain recurrence. Accumulating evidence suggests that immune cells, adhesion molecules, extracellular matrix metalloproteinase and pro-inflammatory cytokines activate/alter peritoneal microenvironment, creating the conditions for differentiation, adhesion, proliferation and survival of ectopic endometrial cells [13–15]. In a study by Tobiume et al., the rAFS score was an independent factor associated with recurrence [16]. Chon et al. reported that dysmenorrhea and ovarian cyst separations significantly affected the postoperative recurrence rate [17]. Selcuk et al. reported that the depth of penetration of the endometrial tissue into the ovarian cyst wall was an independent risk factor for recurrence [18]. Guzel et al. reported that the CA125 level, ovarian cyst size, and history of pelvic surgery affected the recurrence rate [19]. However, it is difficult to compare the results of the above studies due to differences in the study population, the follow-up duration, and the definition of endometrioma and/or endometriosis-related pain recurrence. Notably, most of the above studies reported the endometrioma and/or endometriosis-related pain recurrence rate within 5 years after initial surgery. We have little information about the recurrence rate beyond 5 years after surgery.

**Fig. 1:**
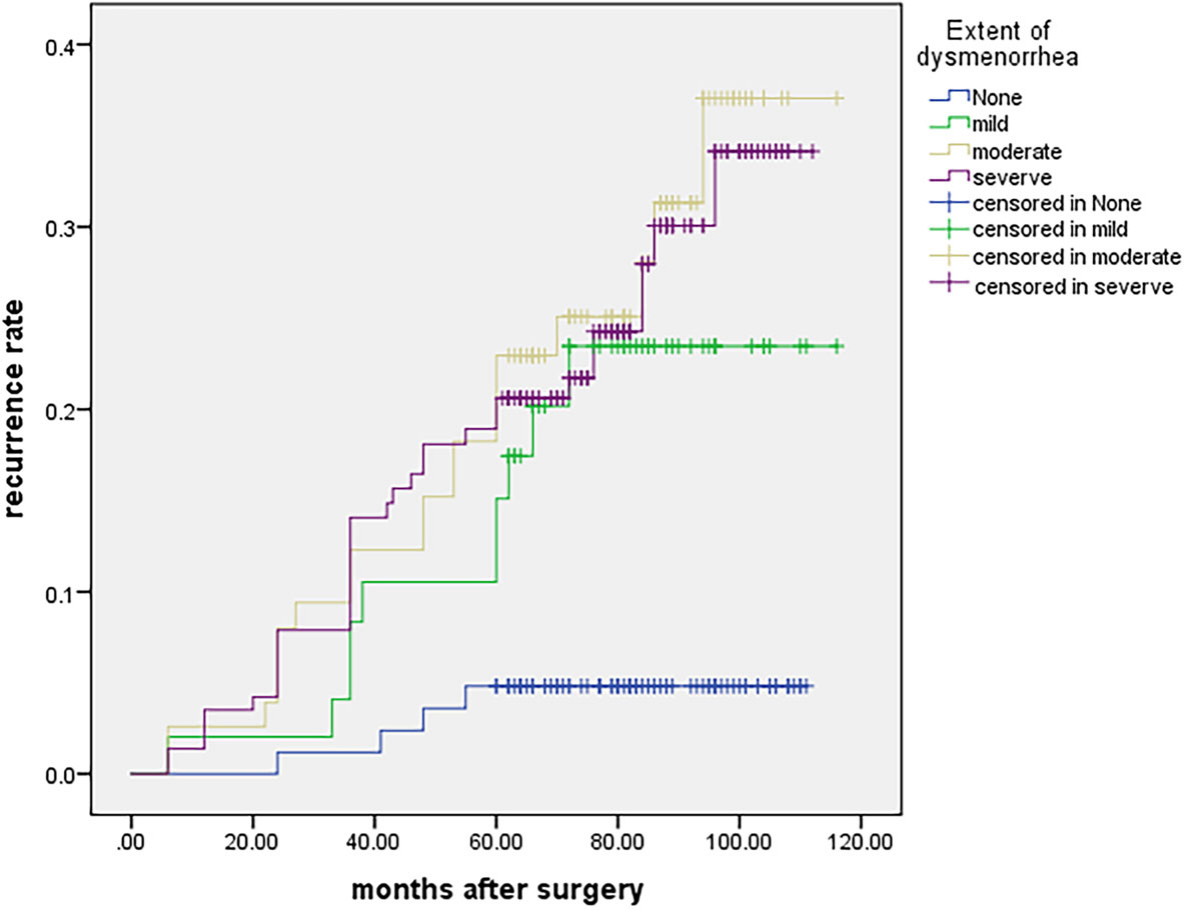
Kaplan-Meier curves presenting the cumulative rate of recurrence according to the severity of dysmenorrhea. There were significant differences between the four groups according to the log-rank test analysis (χ^2^ = 11.487, *p* = 0.001)

**Fig. 2:**
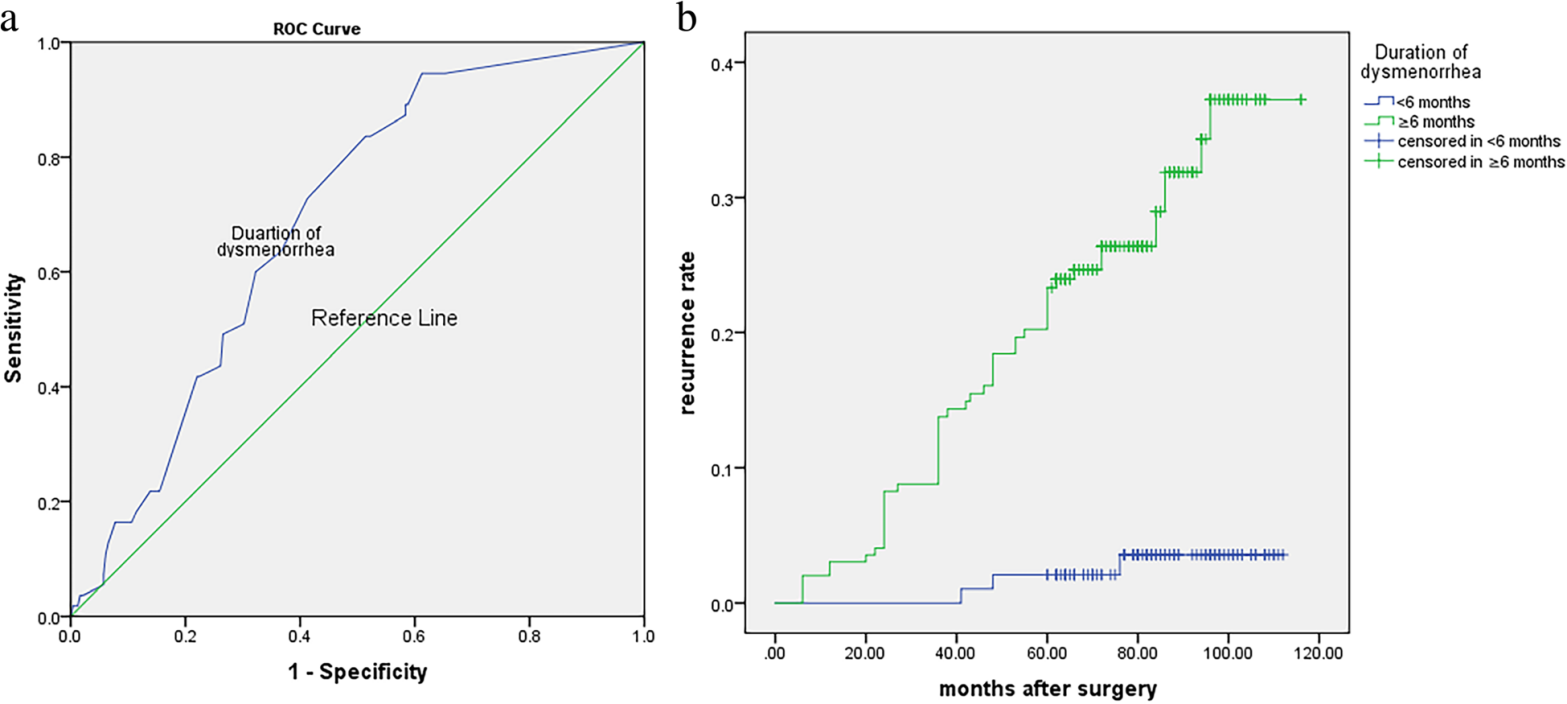
**a** The ROC analysis of duration of dysmenorrhea in recurrent endometrioma patients. Area under curve: 0.689, Cut-off value: 5.5 months; **b** Kaplan-Meier curves presenting the cumulative rate of recurrence according to duration of dysmenorrhea (< 6 months or ≥ 6 months). There were significant differences between the two groups according to the log-rank test analysis (χ^2^ = 22.352, *p* < 0.001)

This study found that the extent of dysmenorrhea and postoperative pregnancy were risk factors according to the multivariate analysis. To avoid multiple collinearity, the presence of dysmenorrhea was not selected for the multivariate analysis. Several studies identified dysmenorrhea as a possible risk factor for endometrioma recurrence [16, 20]. Our study also showed an association between the presence of dysmenorrhea after surgery and endometrioma and/or endometriosis-related pain recurrence in a univariate analysis (Fig. 1). Mechanisms of dysmenorrhea have not been uniformly determined; however, direct and indirect effects of focal bleeding from endometriotic implants, actions of inflammatory cytokines in the peritoneal cavity, and irritation or direct infiltration of nerves in the pelvic floor are causes [21]. It is hypothesized that cyclic recurrent microbleeding within endometriotic lesions with consequent inflammation may be the cause of severe dysmenorrhea among women with endometriosis [22]. Therefore, the longer the disease duration is, the deeper the lesions will infiltrate. In our series, women who suffered dysmenorrhea over 6 months had higher endometrioma and/or endometriosis-related pain recurrence rate (Fig. 2). Over time, most patients with endometriomas had a higher rASRM score and were categorized as having advanced-stage endometriosis. In fact, most of patients (96.9%) in this study demonstrated stage III or IV endometriosis. Adhesions may also cause deep pelvic pain associated with recurrent endometriosis, and postoperative dysmenorrhea also suggests endometrioma recurrence [23]. Because of extensive adhesions and inflammation, incomplete resection can occur in advanced stages of endometriosis. Incomplete surgical removal of endometriomas located on an endometriotic lesion or adhesion only decreases the severity of symptoms because only the removal of visualized cystic lesions or separation of adhesions is performed; Although peritoneal superficial lesions and ovarian endometriomas represent the majority of endometriotic implants within the pelvis, deep infiltrating endometriosis and extrapelvic endometriosis are the most challenging conditions to face off. Despite sometimes medical therapy is enough to reduce symptoms and, in a large number of patients a complete eradication, with nerve-sparing and vascular sparing approach is needed to restore the normal pelvic anatomy and its functions [24, 25]. In our study, all remaining visible endometriotic lesions were excised or fulgurated after the endometriomas were removed. Anatomical restoration was then achieved. However, surgery does not address the underlying mechanisms that are active and driving disease in the pelvic cavity. This naturally leads to recurrence of endometriosis in various forms after a certain period of time.

**Fig. 3:**
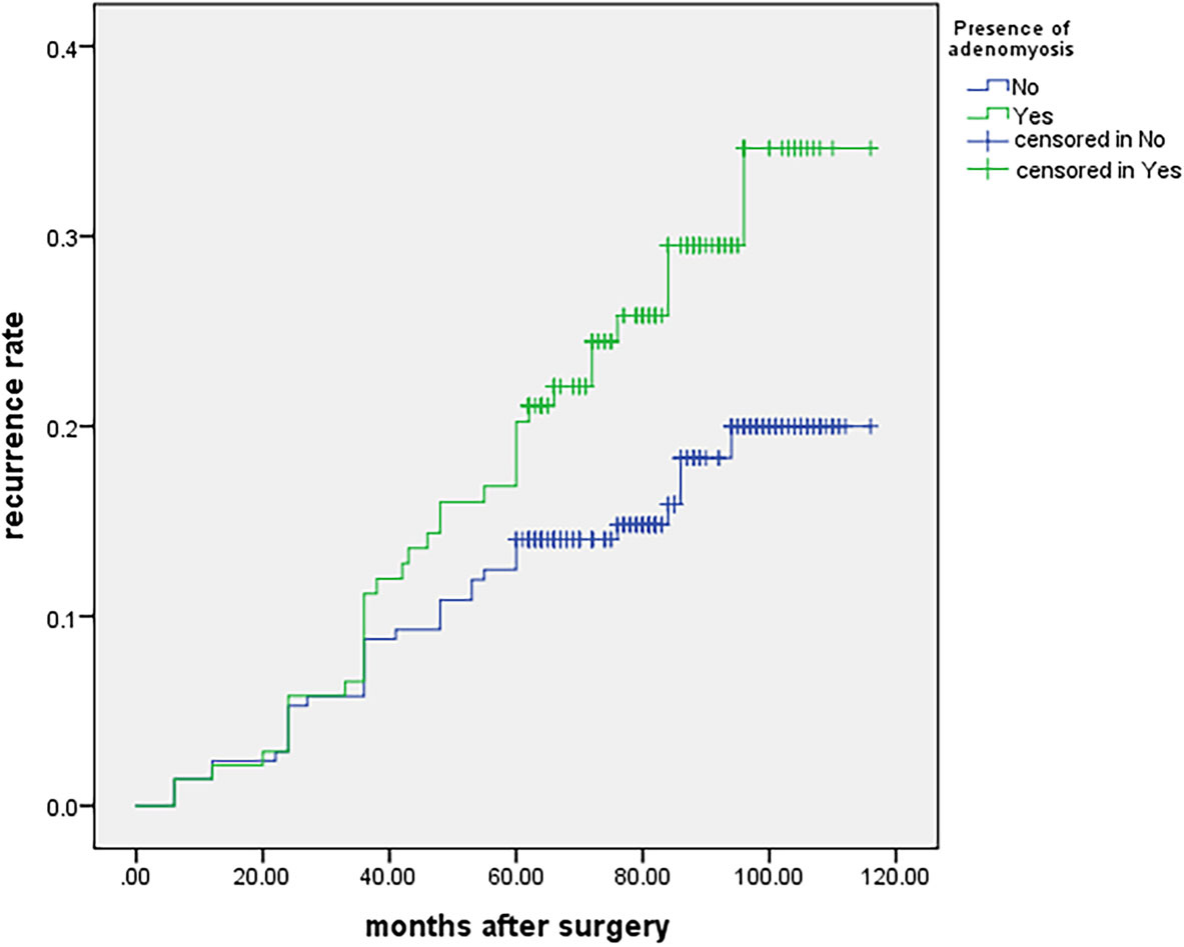
Kaplan-Meier curves presenting the cumulative rate of recurrence according to the presence of adenomyosis. There were significant differences between the two groups according to the log-rank test analysis (χ^2^ = 4.113, *p* = 0.043)

The presence of adenomyosis is another risk factor for endometrioma and/or endometriosis-related pain recurrence. It is well known that there is an overlap in the pathogenesis of endometriosis and adenomyosis [22]. Dior et al. found that sonographic features of adenomyosis may be included as a component of the clinical assessment when attempting to predict the presence of severe endometriosis [26]. We observed a statistically significant correlation between pelvic pain and the presence of adenomyosis (Fig. 3), in agreement with the previously reported results [27, 28]. Dysmenorrhea is a risk factor in the deep adenomyotic process with a high density of endometrial glands in the myometrium [29]. Perello et al. observed a clear trend associating of the presence of dysmenorrhea with adenomyosis, without statistical significance. Among the patients reporting these symptoms, 95.5% described the intensity as severe [30]. Our study showed that the concurrence rate of adenomyosis was up to 50.0% in the recurrence group. Unresolved adenomyosis may consequently lead to pain recurrences after pelvic surgery. Therefore, correct identification of coexisting pathological conditions for DIE and adenomyosis is necessary for the development of effective surgical protocols [30].

**Fig. 4:**
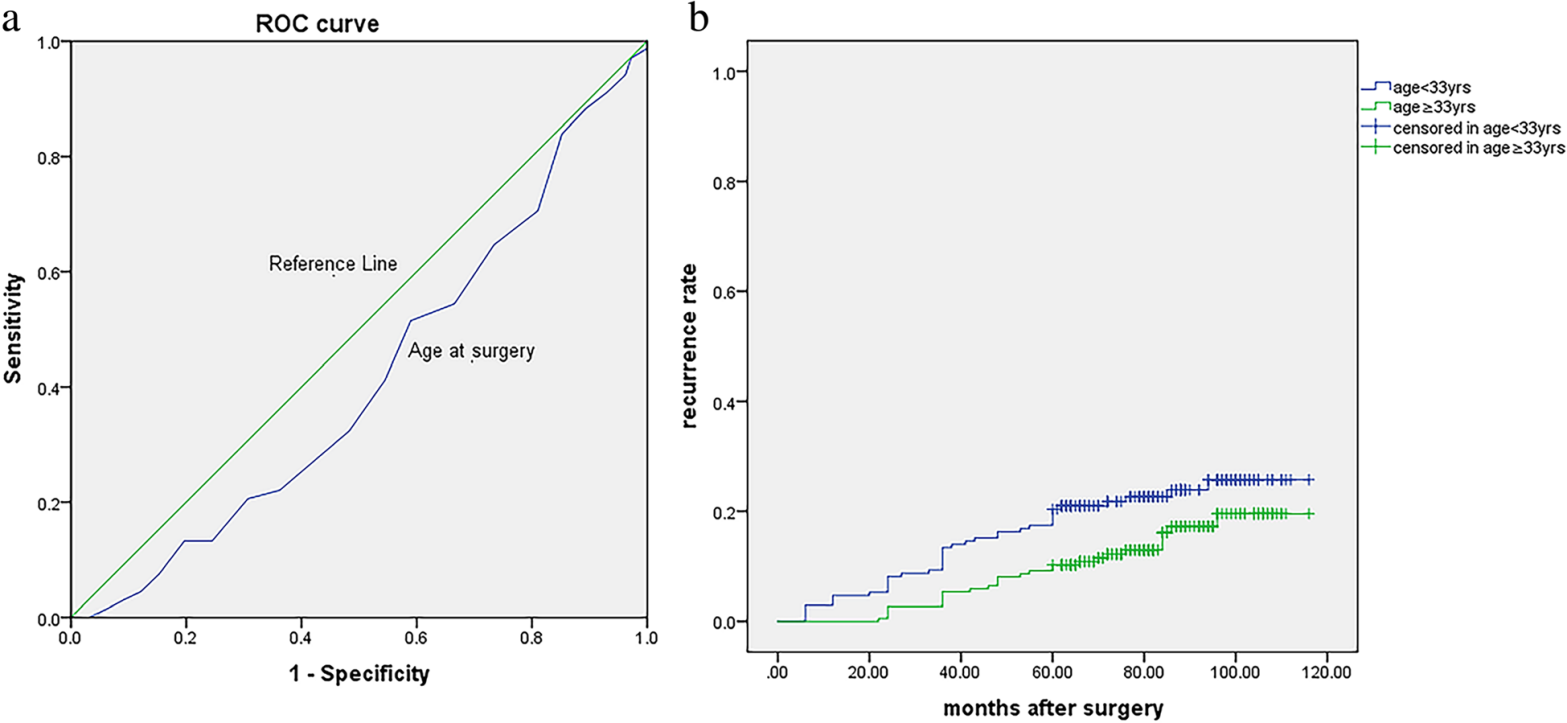
**a** The ROC analysis of age at surgery in recurrent endometrioma patients. Area under curve: 0.413, Cut-off value: 33.5 yrs.; **b** Kaplan-Meier curves presenting the cumulative rate of recurrence according to age at surgery (< 33 yrs. or ≥ 33 yrs). There were significant differences between the two groups according to the log-rank test analysis (χ^2^ = 22.352, *p* < 0.001)

It was reported that age at surgery is associated with the postsurgical endometrioma recurrence rate, and the reason is unclear. We postulate that endometriosis is a hormone dependent disease. Thus, the higher circulation estrogen levels in younger women may produce a more aggressive form of endometriosis; therefore, the younger, the more likely to recur [18]. Seo et al. [31] reported that, at an average follow-up time of 29 months with no postoperative medication, the cumulative endometrioma recurrence rates were 43.3% for patients aged 20–29 years, 22.5% for 30–39 years, and 10.2% for 40–45 years. In our study, the cumulative endometrioma and/or endometriosis-related pain recurrence rates were 24.2% for patients aged 20–30 years, 17.7% for 31–40 years, and 7.9% for 41–45 years, which was consistent with the literature. Our study also found that 33.5 years is a cutoff value in the ROC age analysis (Fig. 4a). Patients who are younger than 33.5 years may have a higher risk for recurrence (Fig. 4b). Our previous study showed that laparoscopic cystectomy for endometrioma with an age greater than 35 years may decrease the remaining ovarian reserve [32]. Therefore, a decrease in the rate of endometrioma and/or endometriosis-related pain recurrence also results in a reduction in ovarian reserve function. 33 to 35 years of age is a “dilemma window”, and we should pay more attention to patients in this age group when performing surgery.

Preventive use of medications after operation is recommended for patients with a high risk of recurrence [33–36]. Many studies have investigated factors determining the recurrence of endometrioma and pain after surgery [16, 19, 20]. The mechanism of oral contraceptive pills (OCPs) reducing ovarian endometrioma recurrence is unclear. OCP increases apoptosis and decreases cell proliferation in eutopic endometria, which can decrease both recurrence from small endometriotic foci not seen at surgery and de novo disease development [33]. Some reports suggest that ovarian endometrioma can develop from ovarian follicles or the corpus luteum and that consequent inhibition of ovulation may decrease the risk of endometrioma development [36–38]. Regardless of the mechanism, the present and previous studies suggest that postoperative medical treatment is known to delay but not completely prevent recurrence. Vercellini et al. [39] reported that postoperative use of GnRHa could only prolong the recurrence interval but could not improve the overall recurrence rate. There is no consensus regarding whether the LNG-IUD could reduce the endometrioma recurrence rate either [40]. Notably, Jee et al. [41] reported that although postoperative GnRH agonist treatment does not reduce objective disease recurrence in stage III/IV disease, GnRHa delays the time of recurrence, as indicated by Vercellini et al. [39]. In our study, we also failed to observe a benefit for postoperative medication in preventing endometrioma and/or endometriosis-related pain recurrence. Patients with advanced-stage endometriosis may prefer to take postoperative medication. This may partially explain why a statistically significant difference was not reached in terms of postoperative medication between patients with and without recurrence. We found that the estimated cumulative endometrioma and/or endometriosis-related pain recurrence rates at 1 to 10 years after surgery were 2.2, 5.3, 9.2, 12.0, 15.4, 16.8, 19.3, 22.5, 22.5, and 22.5%, respectively. These relatively low endometrioma and/or endometriosis-related pain recurrence rates may be due to most patients having postoperative medication applied in our study. The recurrent rate increased yearly until 8 years after surgery.

Most ovarian endometriomas are composed of an extraovarian pseudocystic structure with no cystic wall but are surrounded by fibrosis with underlying ovarian cortical follicles [42]. Therefore, the inner surface of an endometrioma is lined by endometriosis with variable penetration into the surrounding fibrosis. The mean cyst wall thickness varied between 1.2 and 1.6 mm. Endometriosis tissue covers the inner aspect of the cyst for approximately 60% of its surface with a mean depth of penetration of 0.6 mm [43]. With the aging of the endometrioma, infiltration and invasion of the ovarian interstitial, resulting in the incomplete removal of endometriomas [42]. Concordantly, the univariate analysis in our study shows that the duration of dysmenorrhea is significantly associated with endometrioma and/or endometriosis-related pain recurrence (Fig. 2).

It is clear that the prevalence of recurrent endometrioma varies according to whether there is a successful pregnancy after surgical treatment of the endometrioma [44, 45]. Koga et al. demonstrated that patients with postoperative pregnancies had much lower rates of recurrence, which indicates that a subsequent pregnancy may have a protective effect on endometrioma recurrence [46]. In our study, postoperative pregnancies also prevented endometrioma recurrence (OR: 0.649, 95%CI: 0.460–0.914, *p* = 0.013).

The merits of our study were the long time follow-up of more than 5 years, all with detailed record clinical data, the same experienced surgeon, and the large sample size. However, there are some limitations. This was a retrospective study, and it may contain biases with regard to patient characteristics; women with severe forms of endometriosis may prefer to receive preoperative or postoperative medical treatment, and this is a single center study. Recurrent endometrioma was defined by ultrasound as the presence of a persistent ovarian cyst and did not resolve after several successive menstrual cycles. It depends mainly on the skill and experience of radiologists. These limitations may have resulted in an under or over estimation of the associations in our study.

In conclusion, we conducted a long-term follow-up study for more than 5 years. The rate of endometrioma and/or endometriosis-related pain recurrence increased yearly in the first 8 years. Our research revealed that the extent of dysmenorrhea and postoperative pregnancy are independent risk factors for endometrioma and/or endometriosis-related pain recurrence. For patients who have serious dysmenorrhea, we should pay more attention to their risk for endometrioma and/or endometriosis-related pain recurrence and apply individual management to achieve better efficacy.

## Data Availability

The dataset supporting the conclusions of this article is included within the article and its additional files.
